# Long-Term Consequences of Anticancer Therapy—Treatment Complexity and Quality of Life as Determinants of Affective Disorder Phenotypes in Adolescent Cancer Survivors

**DOI:** 10.3390/cancers18111782

**Published:** 2026-05-29

**Authors:** Piotr Pawłowski, Maria Banasik, Mateusz Barłóg, Zuzanna Kwissa-Gajewska, Mikołaj Jeżak, Aneta Kościołek, Emilia Samardakiewicz-Kirol, Małgorzata Mitura-Lesiuk, Marzena Samardakiewicz

**Affiliations:** 1 Department of Psychology, Psychosocial Aspects of Medicine, Faculty of Medicine, Medical University of Lublin, 20-093 Lublin, Poland; mateusz.barlog@umlub.edu.pl (M.B.); marzena.samardakiewicz@umlub.edu.pl (M.S.); 2 Institute of Medical Sciences, University of Applied Sciences in Chełm, 22-100 Chełm, Poland; aneta.kosciolek@umlub.edu.pl; 3 Department of Psychology, John Paul II Catholic University of Lublin, 20-950 Lublin, Poland; maria.banasik@kul.pl; 4 Institute of Psychology, SWPS University, 03-815 Warsaw, Poland; zkwissa@swps.edu.pl; 5 University Clinical Hospital No. 4 in Lublin, 20-090 Lublin, Poland; mikolaj.jezak@usk4.lublin.pl; 6 Department of Nursing Development, Faculty of Health Sciences, Medical University of Lublin, 20-093 Lublin, Poland; 7 Simulation Laboratory for Patient Safety, Department of Medical Education, Medical University of Lublin, 20-093 Lublin, Poland; emilia.samardakiewicz-kirol@umlub.edu.pl; 8 Department of Pediatric Hematology, Oncology and Transplantology, Medical University of Lublin, 20-093 Lublin, Poland; malgorzata.mitura-lesiuk@umlub.edu.pl

**Keywords:** quality of life, cancer survivors, adolescent, treatment, long-term consequences

## Abstract

**Introduction:** Advances in pediatric oncology have transformed cancer into a condition with chronic and long-term developmental consequences. While survival rates have improved significantly, the literature on psychosocial outcomes remains fragmented and inconsistent, with a notable lack of person-centered analyses that account for the heterogeneity of adaptive trajectories. Current evidence fails to explain why survivors with similar clinical profiles exhibit divergent psychological phenotypes, particularly regarding the late effects of multimodal treatments. The aim of this study was to identify heterogeneous psychosocial profiles among adolescent cancer survivors and to examine their associations with treatment complexity and quality of life. **Materials and Methods:** This cross-sectional study included 165 adolescents aged 12–18 years (mean age: 14.64 years) who were in clinical remission following oncological treatment. Standardized assessment tools were used: the Children’s Depression Inventory 2 (CDI-2™) to measure depressive symptoms, the KIDSCREEN-10 index to assess health-related quality of life (HRQoL), and a scale evaluating satisfaction across 14 life domains. Adaptive profiles were identified using a Two-Stage Cluster Procedure, and risk factors were examined using multinomial logistic regression. **Results:** Four clusters were identified in the study population: a depressive–dysphoric profile, an anhedonic-withdrawn profile, a highly adaptive profile, and a mixed (struggling) profile. Treatment complexity was identified as a significant independent predictor of membership in the high-distress (depressive) cluster. While each additional therapeutic modality beyond standard chemotherapy was associated with a markedly increased risk (OR = 8.91; *p* < 0.001), the relatively wide confidence interval (95% CI: 3.27–24.31) suggests that the exact magnitude of this effect should be interpreted with caution. The high lower bound of the interval (3.27), however, strongly supports the directional association of cumulative iatrogenic burden with psychological adaptation. Subjective quality of life functioned as a protective factor against depressive symptoms (OR = 0.57); however, paradoxically, higher self-reported quality of life increased the likelihood of classification into the anhedonic group (OR = 1.81). This divergence between high self-reported HRQoL and social withdrawal potentially suggests a ‘well-being paradox’. It is hypothesized that standard HRQoL instruments may primarily capture physical remission and relief from acute somatic symptoms, potentially masking underlying social–emotional deficits. This suggests that HRQoL scores in survivors should be interpreted with caution and complemented by specific affective screenings. **Conclusions:** The absence of a uniform pattern of psychological response to cancer among adolescent survivors supports the validity of a patient-centered approach. The burden associated with intensive multimodal treatment significantly increases the likelihood of full-syndrome depression during adolescence. Moreover, the identification of a cluster suggestive of anhedonic and socially withdrawn features highlights the limitations of standard screening tools focused solely on the detection of overt sadness. This heterogeneity underscores the need for personalized psycho-oncological care and the implementation of intensified monitoring for patients at high medical risk.

## Simple Summary

Thanks to advances in medicine, the majority of children with cancer can now be cured, which means that cancer increasingly becomes a chronic condition with long-term consequences. The authors of the manuscript examined 165 adolescents aged 12 to 18 years who had completed treatment and were in remission, in order to assess how they cope with returning to everyday life. The study demonstrated that young patients do not respond to the disease in a uniform manner and that four distinct psychological profiles can be identified. The first group represents a “depressive profile”, characterized by predominant sadness and anxiety. The second is an “anhedonic profile”, constituting a form of hidden risk. Adolescents in this group report good overall quality of life yet experience marked difficulties in peer relationships and an impaired ability to experience pleasure. The third group consists of highly adaptive individuals who do not exhibit depressive symptoms, while the fourth is a “fighting profile”, in which adolescents, despite perceiving reduced physical functioning, continue to derive satisfaction from life. The most significant clinical factor associated with poorer well-being was treatment complexity. Each additional therapeutic modality, such as surgery or radiotherapy alongside chemotherapy, was strongly linked to an almost ninefold increase in the likelihood of experiencing elevated depressive symptoms. Notably, sex did not influence adolescents’ adaptation to their new circumstances. Although participants were generally satisfied with their relationships with parents and physicians, they most strongly reported a lack of autonomy and limited opportunities to develop personal interests. The findings indicate that post-treatment care must be tailored to the individual patient. Particular vigilance is warranted for adolescents who have undergone intensive multimodal treatment and for those who withdraw from social life, even if they do not present with overt sadness.

## 1. Introduction

Advances in pediatric oncology have transformed childhood cancer from a fatal disease into a chronic life condition with long-term developmental consequences. As survival rates rise, more children reach adolescence after treatment, often facing persistent physical, psychological, and social challenges rather than full recovery. This shift reflects a biopsychosocial understanding of survivorship, in which medical cure does not necessarily translate into psychosocial well-being [1]. Consequently, research has increasingly moved beyond survival outcomes toward the assessment of long-term functioning and quality of life during critical developmental periods.

Globally, approximately 400,000 new cancer cases are diagnosed annually in individuals aged 0–19 years, with five-year survival exceeding 80% in high-income countries [2,3]. In Poland, survival among children aged 0–14 years reaches 81.2%, resulting in a growing population of adolescent survivors [4]. However, improved survival does not guarantee psychosocial recovery, particularly during adolescence, a developmental stage characterized by identity formation, increasing autonomy, heightened sensitivity to social evaluation, and challenges related to school reintegration and peer functioning [5].

Health-related quality of life (HRQoL) and mental health are therefore central outcomes in pediatric psycho-oncology. HRQoL represents a multidimensional and subjective evaluation of physical, psychological, and social functioning, including school well-being, from the child’s perspective [6]. Evidence regarding the long-term psychosocial sequelae of childhood cancer remains profoundly fragmented. While several large-scale studies report a higher prevalence of fatigue, anxiety, and depressive symptoms among adolescent survivors compared to normative cohorts, other investigations highlight remarkable psychological resilience, with many survivors achieving quality of life scores comparable to healthy peers [7,8,9,10,11]. These inconsistencies are not merely methodological artifacts but likely reflect the inherent heterogeneity of adaptive trajectories during adolescence. Aggregating these diverse experiences into mean group scores often masks distinct clinical subgroups—such as those exhibiting ‘repressed’ or ‘delayed’ distress—thereby limiting the predictive utility of traditional variable-centered analyses. Consequently, there is a critical need to apply person-centered approaches to identify specific psychosocial phenotypes and their unique determinants.

Such heterogeneity underscores the need for person-centered analytical approaches. Cluster analysis allows for the identification of distinct psychosocial profiles based on co-occurring depressive symptoms and satisfaction with different life domains, thereby revealing subgroups with differing psychological needs and levels of functioning. Identifying these profiles may inform more targeted and developmentally sensitive psychosocial interventions. Mapping such profiles also has broader implications for resource allocation and clinical decision-making. Recognizing which late effects and symptoms tend to cluster can inform the design of multidisciplinary follow-up clinics, guide patient education, and support interventions aimed at mitigating long-term morbidity. Ultimately, a comprehensive analysis of these interconnected outcomes in adolescent survivors of childhood cancer can enhance our understanding of treatment-related risks and improve strategies to promote long-term health [12]. To address these gaps, the primary objective of this study was to identify distinct, heterogeneous psychosocial profiles (clusters) among adolescent cancer survivors based on their affective symptoms and satisfaction across multiple life domains. As a secondary objective, we sought to evaluate the predictive role of treatment-related factors (specifically treatment complexity) in determining cluster membership. Additionally, we conducted an exploratory analysis to examine how subjective health-related quality of life (HRQoL) and sociodemographic variables, such as sex and parental education, differentiate these adaptive profiles.

## 2. Materials and Methods

### 2.1. Study Design and Participants

The study employed a cross-sectional design and was conducted among adolescent cancer survivors following childhood malignancy. A purposive sampling strategy was employed to ensure the inclusion of adolescents across the full developmental spectrum of 12–18 years. While this non-probability approach may introduce selection bias, it was necessary to capture a representative range of oncological diagnoses and treatment intensities. To mitigate center-specific variations, a standardized recruitment protocol was enforced across all four clinics. Preliminary analyses (e.g., ANOVA or Kruskal–Wallis) showed no significant differences in the primary outcomes (CDI-2™and HRQoL scores) between the participating centers (*p* > 0.05), justifying the pooling of data.

The research protocol was conducted in accordance with the principles of Good Clinical Practice (GCP) and the Declaration of Helsinki. Ethical approval was obtained from the Bioethics Committee of the Medical University of Lublin (approval no. KE-0254/119/06/2024). Participation was voluntary and anonymous. In compliance with legal requirements for research involving minors, a dual-consent procedure was implemented, including written informed consent from legal guardians (parents) and assent from the adolescent participants themselves. All respondents were informed of their right to withdraw from the study at any stage without providing a reason.

The study was conducted as a multicenter project in outpatient clinics located in Poland (Lublin, Kraków, Gdańsk, and Wrocław) between January and July 2025.

An identical, standardized recruitment protocol was strictly enforced across all four participating centers to identify a clinically homogeneous cohort of adolescents in remission from childhood cancer, minimizing center-specific variations. Specifically, eligible patients and their legal guardians were approached in the clinical waiting area prior to their medical consultation by a designated research psychologist. To minimize selection bias and the risk of perceived coercion, recruitment was not conducted by the patients’ primary treating physicians. The introductory script and consent procedures were strictly uniform across all four sites, ensuring no center-specific variations in the enrollment process. Inclusion criteria comprised a chronological age between 12 and 18 years, histopathologically confirmed diagnosis of childhood cancer treated according to pediatric protocols, and survivorship status defined as the completion of active treatment (chemotherapy, radiotherapy, immunotherapy, or surgery) with confirmed clinical remission. Clinical remission was defined as the complete absence of detectable disease (Complete Remission, CR) verified by the treating oncologist through imaging (CT/MRI) and laboratory markers within 3 months prior to enrollment. A minimum of 6 months since the completion of active therapy was required to distinguish late psychosocial effects from acute treatment-related distress. Pre-existing psychiatric disorders were identified through a multi-source approach: a meticulous review of electronic medical records (EMR), parental reports during the intake interview, and a screening consultation with a designated research psychologist. Additionally, participants were required to demonstrate fluency in Polish to independently complete self-report measures requiring abstract cognitive competence, such as the CDI-2™.

**Figure 1:**
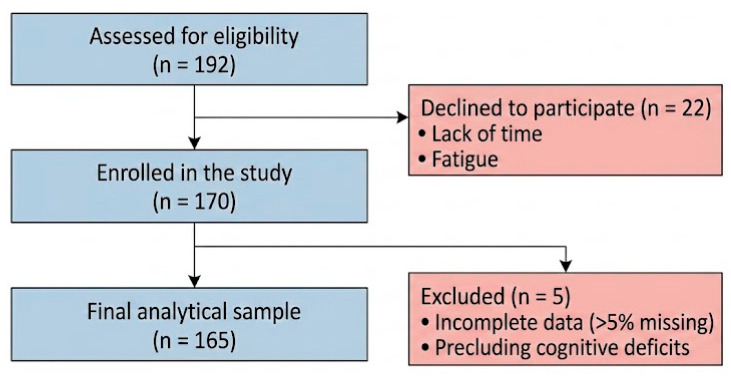
Study flow diagram illustrating the recruitment and selection process of adolescent cancer survivors.

To enhance internal validity and minimize confounding variables, several exclusion criteria were applied. These included a documented history of pre-existing psychiatric disorders (e.g., psychotic or bipolar disorders) diagnosed prior to cancer onset, ensuring that observed symptoms could be interpreted as sequelae of somatic illness. Further exclusion criteria encompassed cognitive or neurodevelopmental deficits precluding independent questionnaire completion, enrollment in palliative care, and incomplete data (defined as >5% missing responses). During the study period, a total of 192 eligible adolescent survivors were identified and approached across all centers. Of these, 22 patients or their guardians declined participation (response rate: 88.5%), primarily citing a lack of time or fatigue. Consequently, an initial sample of 170 patients provided informed consent and were enrolled. Based on the predefined exclusion criteria, 5 individuals were subsequently excluded (due to incomplete data or cognitive deficits precluding independent questionnaire completion), resulting in a final analytical sample of 165 participants. The detailed participant selection process is illustrated in Figure 1.

### 2.2. Research Instruments and Operationalization of Variables

The study protocol comprised standardized psychometric instruments and a review of clinical documentation.

#### 2.2.1. Emotional Functioning and Depression

The severity of depressive symptoms was evaluated using the Polish version of the Children’s Depression Inventory 2 (CDI-2™). This standardized, 28-item self-report instrument allows for a multidimensional assessment of emotional distress in adolescents. Each item is rated on a 3-point scale (0–2), where higher aggregate scores reflect greater symptom intensity. For the purpose of this study, analyses focused on four specific subscales: (A) Negative Mood/Physical Symptoms (assessing dysphoria, sadness, and somatic complaints); (B) Low Self-Esteem/Anhedonia (reflecting deficits in self-concept and a reduced capacity for pleasure); (C) Ineffectiveness (capturing perceived incompetence and lack of agency); and (D) Interpersonal Problems (indicating difficulties in peer relationships and social withdrawal). It is important to clarify subscale B because the official nomenclature in the CDI-2™ manual designates this domain solely as ‘Low Self-Esteem’. However, due to the current lack of a standalone, cross-culturally validated psychometric scale measuring anhedonia for the Polish adolescent population, we purposefully expanded the label for our analytical framework to ‘Low Self-Esteem/Anhedonia’. This theoretical adaptation is justified by the specific item-level content of this subscale, which explicitly assesses core consummatory anhedonic symptoms (e.g., diminished interest, loss of joy, and the inability to experience pleasure). Because identifying latent risk phenotypes was a primary aim of our person-centered analysis, highlighting the anhedonic component structurally embedded within the CDI-2™ was conceptually necessary. Raw scores were converted into standardized T-scores to facilitate comparison with age- and gender-specific national norms. Internal consistency for the scale in the current study was high (Cronbach’s α = 0.86) [13].

#### 2.2.2. Health-Related Quality of Life (HRQoL)

Health-related quality of life (HRQoL) was evaluated using the KIDSCREEN-10 Index. This brief, unidimensional instrument comprises ten standardized items covering physical, psychological, and social dimensions of well-being. Participants respond to each item using a 5-point Likert scale assessing the intensity or frequency of the attribute, with options ranging from ‘not at all’ (1) through “slightly”, “moderately”, and “very”, to “extremely” (5). The questionnaire has been validated for use in the Polish adolescent population and demonstrates high internal reliability (Cronbach’s α = 0.82). Raw scores are transformed into standardized T-scores (Mean = 50, SD = 10), where higher values indicate superior quality of life.

Additionally, the protocol included a single, separate item assessing subjective general health, formulated as “In general, how would you say your health is?” This item was excluded from the total index calculation and analyzed as an independent variable labeled “Self-rated Health”.

#### 2.2.3. Satisfaction with Life Domains and Clinical Variables

**Figure 2:**
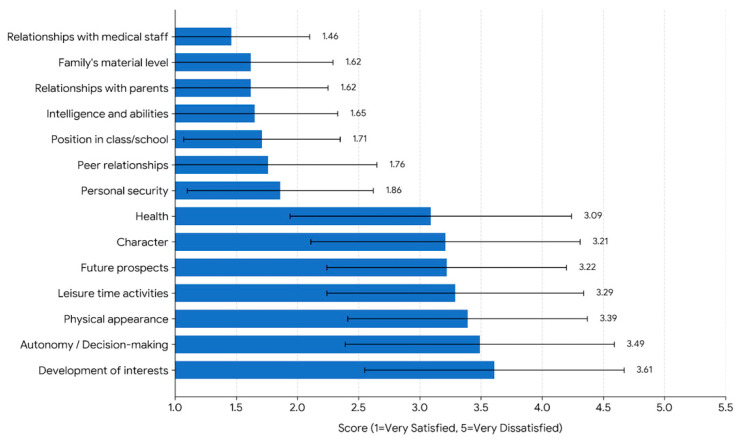
Profile of satisfaction across selected life domains among adolescent survivors (*N* = 165). Mean values and standard deviations are presented. Lower scores indicate higher levels of satisfaction (1 = ‘very satisfied’, 5 = ‘very dissatisfied’).

To capture specific dimensions of psychosocial functioning not fully addressed by general HRQoL measures, an author-constructed (ad hoc) scale assessing satisfaction across 14 life domains was administered. The items were theoretically derived from developmental challenges typical for adolescent cancer survivors. The assessed domains included: health, physical appearance, character, intelligence and abilities, peer relationships, relationships with parents, relationships with medical staff, position in class/school, personal security, autonomy/decision-making, development of interests, leisure time activities, future prospects, and family’s material level. Participants initially rated their satisfaction in each domain on a 5-point Likert scale ranging from 1 (‘very satisfied’) to 5 (‘very dissatisfied’). In the descriptive analysis (e.g., Figure 2), these raw scores were maintained, meaning that lower numerical values correspond to higher levels of perceived satisfaction. While this instrument has not undergone formal psychometric validation, it demonstrated acceptable internal consistency in the current sample (Cronbach’s α = 0.76). To calculate the composite satisfaction index (SATISF) used in advanced analyses, the item scores were reverse-coded so that higher values indicated greater satisfaction, and then the raw scores of all 14 items were summed. To ensure equal weighting and comparability with the CDI-2™ subscales during the subsequent k-means cluster analysis, this raw aggregate score was standardized into a Z-score (Mean = 0, SD = 1) based on the current sample’s distribution.

Medical and sociodemographic data were systematically extracted from clinical records and demographic questionnaires. A comprehensive summary of all collected variables is presented in Table 1. The key independent clinical variable was ‘treatment complexity’ (TREAT), operationalized as an ordinal variable reflecting the number of additional therapeutic modalities applied beyond standard chemotherapy (e.g., radiotherapy, surgery, hematopoietic stem cell transplantation). The operationalization of ‘treatment complexity’ as a cumulative count of modalities (chemotherapy, radiotherapy, surgery, HSCT) follows the Intensity of Treatment Rating Scale (ITR-3) logic. This assumes a cumulative iatrogenic burden where multimodal therapy represents a systemic stressor exceeding that of monotherapy, regardless of the specific biological mechanism of each modality.

To provide a comprehensive biopsychosocial context and to further elaborate on the determinants of adaptation, specific supplementary variables were incorporated into the exploratory models. Clinical metrics included the patient’s chronological age, biological sex, specific oncological diagnosis, history of cancer relapse/recurrence, age at initial cancer diagnosis, and the time elapsed since treatment completion. Socioeconomic status (SES) was operationalized through parental education levels. Importantly, maternal and paternal educational attainments were not aggregated into a single composite index; rather, they were treated as separate categorical variables (coded as: primary/vocational, secondary, and higher education) and entered independently into the inferential analyses.

While specific treatment protocol names were recorded where available, they were not utilized as independent variables in the inferential models due to the high degree of historical heterogeneity and the risk of statistical over-fragmentation. Given that the cohort encompasses various childhood malignancies treated over several years across multiple centers, protocol names do not inherently reflect a standardized level of toxicity or late-effect risk. Consequently, medical burden was operationalized using “treatment complexity”, a cumulative metric reflecting the total number of therapeutic modalities applied. This approach provides a far more robust and statistically viable proxy for the cumulative iatrogenic trauma experienced by the survivor, independent of center-specific or era-specific protocol variations [14,15].

### 2.3. Statistical Procedure

Data were analyzed using IBM^®^ SPSS^®^ Statistics Version 29, with significance set at α = 0.05. Descriptive statistics (M, SD, Mdn, Mo) were computed for demographic and clinical variables.

To identify distinct psychosocial profiles, a structured three-step statistical procedure was implemented:

Step 1: Hierarchical Agglomerative Clustering. To determine the optimal number of clusters, we performed a hierarchical analysis using Ward’s linkage method with squared Euclidean distance as the similarity measure. The analysis was based on five standardized z-scores: the composite satisfaction index (SATISF) and the four CDI-2™ subscales (A, B, C, D). The optimal number of subgroups was determined by inspecting the agglomeration schedule and the resulting dendrogram, which pointed to a four-cluster solution.

Step 2: K-means Iterative Partitioning. To optimize cluster membership and ensure the highest possible internal homogeneity of the groups, we applied the k-means clustering algorithm. The cluster centers identified in the hierarchical stage were used as initial seeds for this iterative process. This Two-Stage Procedure yielded an optimal four-cluster solution with a Silhouette coefficient of 0.58, indicating a ‘good’ and stable cluster structure.

Step 3: Validation and Prediction. To evaluate the internal validity and stability of the cluster solution, a Discriminant Function Analysis (DFA) was employed. Furthermore, to ensure the robustness of the reported 98.8% classification accuracy, a leave-one-out cross-validation procedure was performed, which confirmed the high stability of the identified psychosocial phenotypes. Finally, multinomial logistic regression models were used to identify clinical and demographic predictors of cluster membership, with Cluster 4 as the reference category.

Subsequently, the identified well-being clusters were compared across individual depression and satisfaction with life domains. These comparisons were performed using a series of analyses of variance (ANOVAs) for continuous variables, followed by post hoc Tukey’s HSD tests. In order to determine which demographic, clinical, and quality of life factors independently predicted membership of a well-being cluster, a series of multinomial logistic regressions were performed.

## 3. Results

### 3.1. Characteristics of the Study Group

The analyzed study group comprised 165 participants, after the exclusion of 5 respondents due to incomplete data (2.94% of the original sample). The population was relatively balanced in terms of sex distribution, with a slight predominance of males (52.73%) over females (47.27%). The age distribution was heterogeneous, with the largest proportions represented by 13-year-olds (21.21%) and 18-year-olds (20.00%). The smallest subgroup consisted of 15-year-olds (3.64%). The mean age of the participants was 14.64 years. The detailed clinical and sociodemographic characteristics of the study population, including the distribution of oncological diagnoses, specific treatment modalities, and the treatment timeline, are presented in Table 1.

The clinical sample encompassed a diverse range of childhood malignancies (primarily leukemias, lymphomas, and solid tumors) treated across multiple centers with various contemporary and legacy pediatric oncology protocols. Consequently, the time elapsed since the completion of active treatment varied among participants, reflecting a broad spectrum of the survivorship period.

The diagnostic distribution within the analyzed cohort, characterized by a predominance of acute leukemias (60.6%) and lymphomas (25.5%) alongside a limited representation of solid tumors (3.0%), aligns with the established epidemiological patterns of pediatric oncology in Central Europe. Although this imbalance may constrain the generalizability of the findings to survivors of rare solid tumors or central nervous system malignancies, it ensures robust statistical power for the most prevalent survivor groups. To minimize potential diagnostic bias during cluster identification, ‘treatment complexity’ was utilized as a standardized metric of iatrogenic burden. This approach allows for an analysis that transcends specific tumor biology, focusing instead on the cumulative physiological and psychological impact of multimodal therapy.

In the psychometric domain, the study group demonstrated a distinct polarization in terms of well-being and a high degree of homogeneity in the severity of depressive symptoms (Table 2).

The overall KIDSCREEN-10 quality of life score (M = 35.93; SD = 5.17) demonstrated considerable dispersion. While the most frequent scores (modes) were 38 and 39, the distribution was characterized by a notable negative skew, reflecting a distinct subgroup of patients with significantly lower well-being. Specifically, centile-based analysis relative to the Polish reference population revealed that 32.73% of adolescents (*N* = 54) reported a subjectively perceived quality of life at a critically low level (exceeding two standard deviations below the norm). Although the majority of respondents (68.23%) fell within normative ranges, centile-based analysis relative to the Polish reference population revealed a concerning trend: as many as 32.73% of adolescents (*N* = 54) reported subjectively perceived quality of life at a critically low level (<mean-2 SD).

In contrast to the heterogeneity observed in well-being, CDI-2™ scores demonstrated high homogeneity (M = 56.06; SD = 4.20), with an almost perfect convergence of the mean and median (Mdn = 56.00). Subscale analysis revealed a specific pattern of difficulties. The most burdened domains were CDI-2™ A and CDI-2™ C. The score in subscale A (M = 19.93), with a mode of 21 (close to the maximum possible score), indicates a widespread experience of sadness and dysphoria. Concurrently, a pronounced deficit in the effectiveness domain (M = 15.45; mode = 16) suggests a strong sense of reduced agency among participants. Scores on CDI-2™ B were situated at a moderate level (M = 13.26).

A notable finding was the relatively low score on the CDI-2™ D subscale (M = 8.02; Mdn = 7.00), suggesting that despite pronounced emotional tension and diminished perceived effectiveness, the adolescents less frequently internalized these difficulties in the form of self-aggressive tendencies or persistent negative self-beliefs. To provide a clinically grounded interpretation of depressive symptoms, CDI-2™ T-scores were evaluated against established normative thresholds: scores of 60–64 T represent a ‘high average’ (subclinical) level, 65–69 T indicate ‘slightly elevated’ symptoms, and T ≥ 70 reflect ‘very elevated’ (clinically significant) distress. Although the mean T-score for the total cohort (M = 56.06) fell within the average range, 58.24% of participants (*N* = 96) scored above the subclinical threshold (T > 60) in at least two functional domains. When integrated with the 32.73% of survivors reporting critically low HRQoL (T < 30), these data indicate a substantial subgroup experiencing concentrated impairment. This accumulation of symptoms, particularly in Negative Mood (Subscale A) and Ineffectiveness (Subscale C), identifies over half of the study population as being at elevated clinical risk for affective disorders.

Analysis of the collected research material enabled the identification of a satisfaction profile across 14 key life domains (Figure 2).

Mean scores for the entire study group indicate a pronounced polarization of satisfaction depending on the nature of the assessed domain. The highest ratings were observed for relationships with medical staff (M = 1.46), material standard of living (M = 1.62), and relationships with parents (M = 1.62). The lowest levels of satisfaction concerned opportunities for developing personal interests (M = 3.61) and the ability to make autonomous decisions (M = 3.49). The mean value of the standardized composite satisfaction index (SATISF) was 34.61 (SD = 15.13), indicating a moderate overall level of well-being, accompanied by substantial variability in the personal domain. Overall, the group reported a sense of social security and stability, while simultaneously demonstrating deficits in perceived agency and self-actualization (Figure 2).

### 3.2. Identification of Psychosocial Profiles

For the hierarchical agglomerative clustering, inspection of the agglomeration scree plot and dendrogram revealed a four-cluster solution.

**Figure 3:**
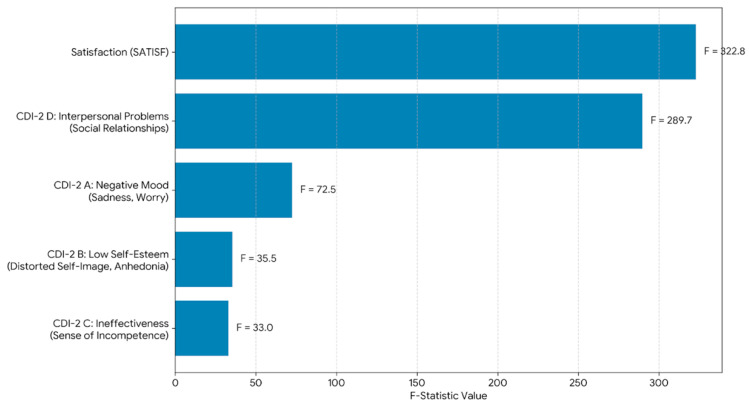
Hierarchy of variable importance in cluster differentiation (F-statistic values determining the strength of cluster differentiation by individual variables).

A k-means clustering analysis yielded a four-cluster solution, distinguishing subgroups with discrete well-being profiles. Analysis of variance (ANOVA) confirmed that all included variables significantly differentiated the identified clusters (*p* < 0.001). The strongest discriminators were satisfaction with Life Domains (F = 322.8) and the CDI-2™ D subscale (F = 289.7) (Figure 3).

**Figure 4:**
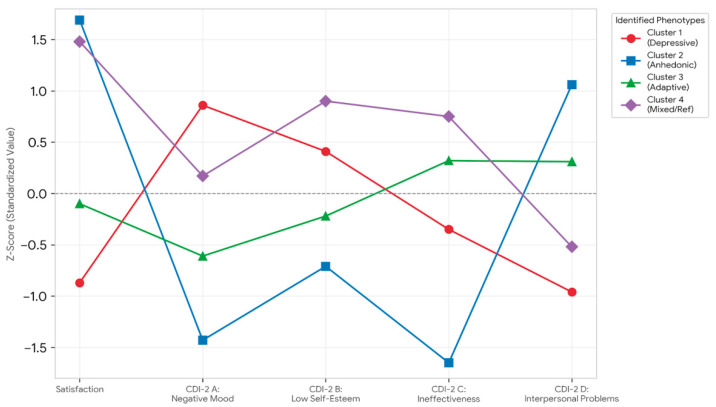
Psychological profiles of distinct clusters (standardized results) (The graph shows the average Z-score results for each variable. A value of 0 represents the average for the entire study group). Labels such as ‘Depressive’ or ‘Anhedonic’ are used here as descriptive, operational designations of symptom clusters and do not imply formal clinical diagnosis.

The DFA yielded two discriminant functions which explained 55.7, 34.3 and 10.0% of the variance, respectively (Wilks’ lambda = 0.013, χ^2^ (15) = 693.6, *p* < 0.001; Wilks’ lambda = 0.095, χ^2^ (8) = 375.2, *p* < 0.001; Wilks’ lambda = 0.468, χ^2^ (3) = 121.1, *p* < 0.001), and the significant results indicated that the corresponding function explained the group membership well. The 98.8% classification accuracy was further confirmed by leave-one-out cross-validation, which yielded an identical result, demonstrating the exceptional robustness of the model. Well-being profiles of four clusters are shown in Figure 4.

For the purpose of this study, the identified clusters were assigned descriptive labels (e.g., ‘depressive’, ‘anhedonic’). It is important to emphasize that these terms refer to statistical phenotypes based on self-reported symptom patterns and do not constitute formal psychiatric diagnoses. These labels are utilized hereafter as operational shorthand to facilitate the interpretation of the diverse adaptive profiles identified in the adolescent survivor population.

Cluster 1: “depressive-dysphoric”—This cluster is characterized by the highest level of negative mood accompanied by low satisfaction. Patients in this group present a classical depressive profile dominated by sadness, anxiety, and low mood. These symptoms are accompanied by moderate relational difficulties (CDI-2™ B = 0.41). Notably, the level of anhedonia is relatively low (CDI-2™ D = −0.96), suggesting a preserved capacity to experience pleasure that is nevertheless overshadowed by intense sadness. This cluster represents a group of overt psychological distress.

Cluster 2: “anhedonic-withdrawn”—This group is characterized by the lowest satisfaction of all clusters (SATISF = −1.10), while paradoxically obtaining very low scores on negative mood (CDI-2 A = −1.43) and perceived inefficacy (CDI-2™ C = −1.65). The sole indicator of pathology in this cluster is markedly elevated interpersonal disturbance (CDI-2™ D = 1.06). This profile represents a group at latent psychological risk.

Cluster 3: “highly adaptive”—This cluster demonstrates the highest level of satisfaction (SATISF = 1.69). Negative mood is reduced (CDI-2™ A = −0.61), while the remaining indicators (CDI-2™ B, CDI-2™ C, CDI-2™ D) remain close to the mean. This group represents patients who have adapted most effectively to the experience of illness, maintaining high psychological well-being and exhibiting no clinically significant depressive symptoms.

Cluster 4: “striving/fighting”—Participants in this cluster report high satisfaction (SATISF = 1.48) despite experiencing marked functional difficulties, including a high sense of inefficacy (CDI-2™ C = 0.75) and interpersonal problems (CDI-2™ B = 0.90). Despite objective challenges, these individuals are able to derive satisfaction from life and retain the capacity to experience pleasure (CDI-2™ D = −0.52).

A profile analysis indicated significant differences between the profiles of the four-cluster groups across the five well-being domains. The groups differed on average well-being levels, as indicated by a between-subject test, F(3, 161) = 152.95, *p* < 0.001, ηp^2^ = 0.74. The flatness test revealed the presence of significant differences between domains within the groups, F(1.12, 180.90) = 1232.68, *p* < 0.001, ηp^2^ = 0.88. The group standardized means on each well-being and the differences between groups can be observed in Table 3.

### 3.3. Predictors of Cluster Membership

To identify predictive factors determining membership in the identified clusters, a multinomial logistic regression analysis was conducted. Cluster 4, characterized by high satisfaction despite moderate difficulties, was selected as the reference category, allowing for the estimation of relative risk in comparison with a group demonstrating relatively good adaptation. The following predictors were entered into the model: (1) clinical variable reflecting treatment complexity; (2) cancer type; (3) sex; and (4) subjective quality of life (KIDSCREEN-10) (Table 4).

In the preliminary multinomial regression models, the type of cancer diagnosis emerged as a statistically significant predictor of the “depressive-dysphoric profile” (OR = 0.42; 95% CI: 0.26–0.66; *p* < 0.001). However, the distribution of diagnoses within the cohort was highly skewed, dominated by acute leukemias (60.6%) and lymphomas (25.5%), with a marginal representation of solid tumors (3.0%). While ‘type of cancer’ was retained in the model as a baseline control variable (Table 4), its specific estimates are not the primary focus of clinical interpretation due to the risk of sparse data bias. Instead, our functional analysis prioritizes “treatment complexity” as the statistically robust metric of cumulative iatrogenic burden.

Comparison of the poorest-functioning group (Cluster 1) with the reference group revealed a strong association with medical variables and quality of life. Treatment complexity emerged as the strongest independent risk factor. Each additional therapeutic modality beyond standard chemotherapy was associated with almost ninefold higher odds of membership in the depressive cluster (OR = 8.91; 95% CI: 3.27–24.31; *p* < 0.001). While the wide confidence interval (95% CI: 3.27–24.31) suggests lower precision regarding the exact magnitude of this effect due to subgroup sample sizes, the high lower bound strongly confirms the significant directional risk associated with multimodal therapy. A statistically significant protective effect was also observed. Cancer diagnosis type was identified as a significant predictor (Wald = 13.74; *p* < 0.001), indicating that disease biology independently modifies psychological outcomes. Certain diagnoses were associated with a reduced risk of belonging to the depressive cluster relative to the reference group (OR = 0.42; 95% CI: 0.26–0.66). A strong protective effect was also observed for quality of life. Higher scores on the quality-of-life scale were associated with a lower likelihood of membership in the depressive phenotype (OR = 0.57; 95% CI: 0.46–0.71; *p* < 0.001). Sex did not significantly differentiate the risk of belonging to this cluster (*p* = 0.595), suggesting that mechanisms of psychological decompensation under intensive treatment are independent of patient sex.

Analysis for Cluster 2 revealed a distinct pattern of determinants, suggesting a different psychopathological mechanism than that observed in the depressive cluster. In contrast to Cluster 1, a higher KIDSCREEN-10 score acted as a positive predictor in this group. An increase in perceived quality of life raised the odds of belonging to the anhedonic cluster by 81% relative to the reference group (OR = 1.81; 95% CI: 1.33–2.47; *p* < 0.001). In conjunction with low satisfaction and high social withdrawal, this finding suggests that high scores on a general HRQoL index like the KIDSCREEN-10 may primarily reflect the patients’ physical recovery and relief from somatic symptoms following treatment, rather than their actual psychosocial well-being and hedonic capacity. The effect of cancer type on anhedonic presentation approached statistical significance (*p* = 0.054; OR = 0.67), suggesting a trend whereby biological factors play a contributory, though not dominant, role. Treatment complexity was not a significant predictor in this cluster (*p* = 0.393), indicating that anhedonia and withdrawal are not a direct consequence of medical burden, but may instead reflect personality-related factors or alternative coping mechanisms.

Comparison of the best-functioning group (Cluster 3) with the reference group revealed no significant differences across the analyzed variables. Neither “treatment complexity” (*p* = 0.862), quality of life (*p* = 0.938), nor sex (*p* = 0.599) significantly differentiated these groups. This suggests that the distinction between “good adaptation” (Cluster 4) and “very good adaptation” (Cluster 3) is not attributable to external (treatment-related) or demographic factors, but rather to psychological resources not captured in the regression model, such as resilience or social support. Only a statistical trend was observed for cancer type (*p* = 0.068), further supporting the notion that exceptional adaptation is less dependent on medical characteristics of the disease.

In accordance with clinical significance and to further elaborate on the determinants of the identified phenotypes, an exploratory trend analysis was conducted using multinomial logistic regression (with Cluster 4 as the reference). Given the preliminary nature of these associations and the constraints of subgroup sample sizes, these results should be interpreted as hypothesis-generating rather than confirmatory (Table 5.)

While the time elapsed since treatment completion did not significantly differentiate cluster membership (*p* > 0.05), age at diagnosis emerged as a significant predictor. Older age at initial diagnosis was associated with a lower likelihood of classification into the “depressive profile” (Cluster 1: OR = 0.62; 95% CI: 0.44–0.87; *p* = 0.007).

Regarding socioeconomic trends, parental education levels demonstrated complex associations with maladaptive phenotypes. Higher paternal education marginally increased the odds of membership in the “depressive profile” (Cluster 1: OR = 2.20; 95% CI: 1.03–4.72; *p* = 0.042), whereas higher maternal education was significantly associated with the “anhedonic-withdrawn profile” (Cluster 2: OR = 3.89; 95% CI: 1.22–12.41; *p* = 0.022).

Robust social support, specifically satisfaction with peer relationship, acted as a profound protective factor, drastically reducing the risk of membership in the depressive cluster (OR = 0.003; *p* < 0.001). Even accounting for sample size limitations, these exploratory trends underscore that both subjective somatic burden and social anchoring heavily modify the psychological late effects of cancer treatment.

## 4. Discussion

The findings of the present study indicate clear heterogeneity in health-related quality of life (HRQoL) among adolescents who experienced cancer during childhood. Although the majority of participants functioned within normative ranges, a substantial proportion of the sample (32.73%) exhibited a relatively lower level of subjective well-being. This observation aligns with the heterogeneity of survivors reported in the literature and underscores the need to identify factors responsible for this variability.

Previous research on psychosocial adjustment following childhood cancer has yielded inconclusive results. Some analyses suggest that many survivors achieve levels of psychological functioning comparable to those of their healthy peers, whereas other studies, particularly those focusing on quality of life, document persistent deficits in HRQoL [16,17,18,19]. These discrepancies have been attributed, among other factors, to methodological differences, the selection of reference groups, and the aggregation of individuals at different developmental stages within analyses [20,21]. In this context, increasing emphasis has been placed on person-centered approaches, which allow for the examination of diverse patterns of adaptation rather than relying solely on average levels of functioning [22].

In line with this perspective, the present study employed cluster analysis and identified four distinct psychosocial profiles. The resulting clusters can be interpreted as different configurations of adaptation coexisting within a single clinical population.

The highly adaptive profile, characterized by low levels of depressive symptoms and high life satisfaction, is consistent with prior reports indicating that intact psychosocial functioning represents one of the observable patterns among young cancer survivors [23]. Other studies of individuals who survived childhood cancer similarly confirm the presence of a substantial subgroup that does not exhibit clinically significant psychological problems [24,25]. In the present study, membership in this profile was not significantly associated with clinical variables, which may suggest a relative independence of certain adaptive processes from medical disease parameters. Comparable observations have been reported previously, showing no clear relationship between treatment complexity and psychological outcomes among well-functioning survivors [26].

A different pattern emerged in the depressive-dysphoric profile. In this group, treatment complexity was a significant predictor of membership in a cluster characterized by globally reduced psychological functioning, with a greater number of therapeutic modalities being associated with a significantly higher likelihood of belonging to this cluster. This finding is consistent with the literature indicating that cumulative treatment burden and long-term treatment sequelae may increase vulnerability to psychosocial difficulties [27,28]. At the same time, higher subjective quality of life significantly reduced the risk of membership in this phenotype, supporting its potential protective role in survivors’ psychological functioning. Additionally, the type of oncological diagnosis differentiated the risk of belonging to the depressive profile, which is consistent with large cohort studies demonstrating that cancer type is one of the factors influencing psychosocial functioning among individuals treated for cancer in childhood [29].

In addition to the two relatively distinct adaptive profiles, the analysis also revealed two patterns with a more complex psychometric structure. The anhedonic-withdrawn profile was characterized by very low life satisfaction accompanied by relatively low levels of reported negative mood and feelings of inefficacy, alongside elevated interpersonal difficulties. This configuration corresponds with broader findings indicating the heterogeneous nature of psychological distress in the population of childhood cancer survivors. Longitudinal analyses conducted within the Childhood Cancer Survivor Study have shown that trajectories of depressive symptoms, anxiety, and somatization may develop differently over time, suggesting relative autonomy among different dimensions of emotional functioning in this population [30]. The absence of a significant association between this profile and treatment complexity, as well as only a marginal effect of cancer type, further suggests that this pattern is not a simple consequence of medical burden and may instead be related to more complex psychosocial mechanisms.

From a clinical perspective, it is particularly important that such a pattern may remain undetected in standard screening procedures that focus primarily on symptoms of low mood, thereby potentially increasing the risk of overlooking a subset of young survivors in need of support. This issue is consistent with observations from studies on the organization of survivorship care. In a systematic review of mental health screening in this population, Holmer et al. (2023) demonstrated that although international guidelines recommend routine monitoring of mental health during follow-up visits, in clinical practice some patients with clinically significant symptoms remain without specialist support, and screening procedures are often unsystematic [31]. This suggests that profiles with less typical emotional manifestations, those not primarily expressed through overtly reported low mood, may be particularly prone to being overlooked.

The “fighting profile” comprised respondents who reported high life satisfaction and preserved capacity to experience positive emotions despite co-occurring elevated feelings of inefficacy and interpersonal difficulties. This pattern indicates that the presence of functional problems does not necessarily lead to globally reduced psychological well-being. This phenomenon corroborates earlier findings from research on resilience in survivor populations, showing that a substantial proportion of young individuals maintain good psychosocial functioning despite objective health-related burdens [32].

Regarding social determinants, satisfaction with peer relationships appeared as a profound protective factor. However, the extreme magnitude of this association (OR = 0.003) in the exploratory model must be interpreted with significant caution. Such strong effect sizes may be inflated by the relatively small number of participants in specific cluster strata and likely reflect a high degree of overlap between social withdrawal symptoms and the predictive variables used. Future longitudinal studies with larger cohorts are essential to determine whether this association represents a stable protective mechanism or a statistical artifact of the symptom-overlap between depression subscales and peer satisfaction metrics.

The absence of significant differences related to biological sex suggests that psychosocial adaptation processes following oncological treatment may have a relatively universal character in this population, which is consistent with some previous studies emphasizing the predominant role of individual and environmental factors over demographic variables [33].

Overall, the findings indicate that the psychosocial sequelae of childhood cancer are multidimensional and manifest as diverse configurations of functioning, partially associated with both medical factors and subjective assessments of quality of life. The application of a person-centered approach enabled the capture of this heterogeneity and highlights the need for more individualized psychosocial monitoring in this population.

However, the interpretation of these predictive models must be contextualized within the broader biopsychosocial environment. It is important to clarify that comprehensive systemic and familial factors were not directly tested as primary predictors in our main regression analysis. While our primary models robustly identified treatment complexity and subjective quality of life as significant determinants, the adaptive trajectories we observed are undoubtedly influenced by unmeasured or only exploratorily assessed systemic confounders. For instance, robust community support networks, specific family dynamics, or high socioeconomic status (SES) likely serve as critical external buffers, potentially explaining the resilience of adolescents in the ‘highly adaptive’ or ‘mixed-adaptation’ profiles despite objective medical burdens. Furthermore, the time elapsed since treatment completion likely moderates the intensity of these psychosocial sequelae, as the transition from active patient to long-term survivor presents distinct, time-dependent developmental challenges. Future clinical models must integrate these systemic, familial, and temporal variables to fully map the complex survivorship experience.

The preliminary observation of the ‘anhedonic-withdrawn’ profile warrants methodological caution, as anhedonia was assessed indirectly in this study. Due to the lack of a cross-culturally validated, standalone anhedonia scale for the Polish adolescent population, we relied on the standardized subscales of the CDI-2™ (Subscale B and Subscale D) as clinical proxies. While this approach successfully identified a subgroup with marked social and consummatory deficits, it cannot capture the multidimensional nuances—such as the distinction between anticipatory and consummatory anhedonia—provided by dedicated reward-processing instruments. Consequently, this finding is strictly hypothesis-generating and underscores the need for future validation using specific clinical tools [34,35].

Study Limitations

The primary limitation of this study is its cross-sectional design, which precludes causal inference regarding the directionality of relationships. Specifically, it remains undetermined whether the anhedonia observed in Cluster 2 represents a stable trait or a state-dependent response, and the temporal progression of symptoms cannot be assessed. While the total sample size (*N* = 165) met the requirements for cluster analysis, stratification resulted in relatively small subgroups (n = 34–45). This reduced statistical power and precision, as evidenced by wide confidence intervals for the “treatment complexity” variable. Furthermore, although the study was conducted as a multicenter project, the use of purposive sampling exclusively within selected Polish outpatient clinics limits the external validity and generalizability of the findings to survivor populations treated under different healthcare systems or alternative protocols. Specifically, sampling from patients attending scheduled follow-up visits introduces a potential selection bias; survivors experiencing severe social withdrawal or profound anhedonia might be less likely to attend these voluntary appointments or consent to psychological evaluation, which could lead to an underestimation of the most severe psychosocial deficits.

Methodologically, the study relied exclusively on self-report measures, introducing potential common method variance and social desirability bias. Phenotypes were identified based on symptom severity rather than structured clinical interviews (e.g., Structured Clinical Interview (SCID), Mini-International Neuropsychiatric Interview (MINI)); thus, they do not constitute formal nosological diagnoses. Furthermore, the “anhedonic-withdrawn” profile was identified based on the validated subscales of the CDI-2™ and life satisfaction metrics, as currently, no standalone, psychometrically validated scale for anhedonia exists for the Polish adolescent population. Future research should prioritize the cross-cultural adaptation of dedicated reward-processing instruments to further refine the clinical contours of this profile.

The use of an ad hoc satisfaction scale, although theoretically grounded and demonstrating acceptable reliability (α = 0.76), represents a methodological limitation as the instrument has not undergone formal psychometric validation in a larger normative population.

A further methodological limitation of our profile analysis is the inherent circularity in utilizing the same psychometric variables (CDI-2™ subscales and SATISF) both to identify the clusters and to subsequently describe their functional differences (e.g., via ANOVA and Discriminant Function Analysis). While cross-validation confirmed the high stability of group assignment, the descriptive differences between clusters on these specific domains should be interpreted cautiously as characteristics of the algorithm’s partition, rather than as independent, confirmatory findings.

Furthermore, the skewed diagnostic distribution, particularly the low number of patients with solid tumors, constitutes a limitation. Different malignancies and their specific treatment protocols (e.g., extensive surgeries or localized radiotherapy) may result in distinct late-effect profiles that were not fully captured in this study due to the dominance of hematological malignancies. Future research should aim for a more stratified recruitment to validate these psychosocial phenotypes across a broader spectrum of oncological diagnoses.

A limitation of our predictive model is the wide confidence interval observed for the ‘treatment complexity’ variable. This likely reflects the relatively small size of specific treatment-modality subgroups within the identified clusters. Consequently, while the association between multimodal therapy and psychological distress is statistically significant and clinically plausible, future large-scale studies are required to more precisely calibrate this risk.

Finally, while our exploratory trend analysis incorporated external variables such as parental education, age at diagnosis, and somatic symptoms, the stratification of our sample (*N* = 165) into specific clusters resulted in relatively small subgroups. This limits the statistical power required for highly complex, multifactorial regression modeling without the risk of overfitting. The exploratory findings presented in Table 5 regarding parental education and peer support should be considered preliminary. The stratification of the sample (*N* = 165) into four clusters resulted in small sub-cells, which increases the risk of inflated odds ratios and limits the statistical power required for complex multifactorial modeling. An inherent methodological constraint of the present analysis is the skewed distribution of primary oncological diagnoses. While initial regression models indicated an association between specific cancer types and the “depressive-dysphoric profile”, partitioning such a disproportionate categorical variable across four distinct clusters introduces the risk of sparse data bias (empty or low-frequency cells), which can artificially distort standard errors. Consequently, we approached these specific estimates with considerable caution, refraining from attributing distinct adaptive trajectories solely to the biological taxonomy of the tumor. A related limitation is the omission of specific treatment protocol names in the inferential models. Although protocols dictate the intensity of therapy, their rapid evolution in pediatric oncology makes historical comparisons based solely on nomenclature highly problematic and prone to severe historical confounding bias. We mitigated this by utilizing “treatment complexity” as a standardized proxy for cumulative iatrogenic burden. Consequently, the present findings should be considered an essential pilot investigation. They establish a foundational framework that paves the way for more expansive, targeted research in this domain. This is particularly crucial given the pronounced scarcity of comprehensive epidemiological and psychosocial data regarding childhood cancer survivors in Poland. Future large-scale, multi-center studies utilizing national registries are necessary to provide the statistical robustness required to model the precise impact of granular historical treatment protocols, detailed socio-economic indices, and exact times since treatment completion on long-term psychosocial phenotypes.

Practical Implications and Directions for Future Research

The findings of this study demonstrate a significant association between treatment complexity and the presence of affective symptoms. Adolescents who have undergone multimodal therapeutic protocols exhibit a higher likelihood of membership in the high-distress cluster. However, given the cross-sectional nature of the present data, these results must be interpreted as identifying co-occurring risk factors rather than establishing a direct causal pathway. While it is plausible that cumulative iatrogenic burden contributes to psychological vulnerability, the observed relationships may also be moderated by unmeasured factors, such as individual biological susceptibility or pre-existing resilience levels. Consequently, survivors exposed to intensive treatments should be prioritized for long-term monitoring, as these clinical characteristics serve as robust markers of potential psychosocial risk. Furthermore, the protective role of subjective quality of life highlights the necessity of integrating psychosocial programs, focusing on school reintegration and autonomy, into standard oncological care as a preventive buffer against long-term sequelae.

The observation of a profile suggestive of anhedonic features, characterized by reduced satisfaction without overt sadness, exposes the limitations of screening tools focused exclusively on negative affect. Clinical assessment must therefore expand to evaluate hedonic capacity and social functioning, particularly in patients presenting with withdrawal despite adequate reported quality of life. Consequently, routine clinical evaluations must incorporate a comprehensive psychosocial anamnesis that accounts for systemic and sociodemographic determinants, such as the patient’s age at diagnosis, parental education levels, and the quality of their social support network. Identifying these underlying vulnerabilities during the initial clinical interview is crucial for fully contextualizing the survivor’s functioning. The present findings suggest that future interventional research should explore the efficacy of stratified therapeutic approaches. For instance, evaluating whether patients with high-distress features respond better to standard cognitive-behavioral interventions, while those with anhedonic-withdrawn profiles might benefit more from behavioral activation strategies or social skills training, represents a promising direction for clinical trials.

## 5. Conclusions

1. The absence of a uniform adaptive pattern in adolescent cancer survivors validates the utility of a person-centered approach in uncovering high-risk subgroups that may remain undetected by standard aggregate analyses.
2. The analyses identified a cluster suggestive of anhedonic and socially withdrawn features, characterized by deficits in life satisfaction and interpersonal functioning without overt sadness. This hypothesis-generating finding highlights a potential risk of diagnostic oversight and the need for comprehensive screening.
3. In the current sample, treatment complexity demonstrated the strongest observed association with adverse psychological outcomes; multimodal therapy was strongly linked to a higher prevalence of high-distress symptoms, underscoring the clinical relevance of cumulative iatrogenic burden.
4. Subjective quality of life was associated with a lower risk of classical depressive symptoms, highlighting the value of monitoring overall well-being as a potential marker of psychological resilience.
5. Mechanisms of psychological adaptation to cancer and its treatment appeared to operate universally in this cohort, showing no significant differentiation based on the adolescent’s biological sex.
6. These findings highlight the potential value of personalizing psychosocial follow-up care and tailoring support to specific functional profiles. However, the efficacy of specific targeted interventions and the broader clinical implications of these phenotypes require confirmation through longitudinal research and externally validated studies.

## Figures and Tables

**Table 1:** Clinical and therapeutic characteristics of the study population (*N* = 165).

Clinical Variable	Total Cohort *N* = 165 (%)
Oncological Diagnosis *	
Acute Leukemias	100 (60.6%)
Lymphomas (Hodgkin’s, Non-Hodgkin’s)	42 (25.5%)
Solid tumors (Neuroblastoma, Sarcomas)	5 (3.0%)
Unknown/Lack of medical records	18 (10.9%)
Treatment Modalities **	
Chemotherapy (Total)	165 (100.0%)
Chemotherapy only (Monotherapy)	100 (60.6%)
Chemotherapy + Radiotherapy	21 (12.7%)
Chemotherapy + Surgery	18 (10.9%)
Chemotherapy + Hematopoietic Stem Cell Transplantation (HSCT)	33 (20.0%)
Cancer Relapse/Recurrence	
No	114 (69.1%)
Yes	51 (30.9%)
Age at Diagnosis (years)	
0–2	18 (10.9)
3–5	24 (14.5)
5–7	55 (33.3)
8–12	25 (15.2)
12–15	30 (18.2)
16–18	13 (7.9)
Time Since Treatment Completion (years)	
0–2	55 (33.3)
3–5	26 (15.8)
5–7	54 (32.7)
8–10	5 (3.0)
>10	25 (15.2)
Socioeconomic and Psychosocial Characteristics	
Father’s Education Level	
Primary/Vocational	19 (11.5)
Secondary	65 (39.4)
Higher	67 (40.6)
Unknown	14 (8.5)
Mother’s education level	
Primary/Vocational	4 (2.4)
Secondary	66 (40.0)
Higher	76 (46.1)
Unknown	19 (11.5)
Peer Relationships (Satisfaction)	
Very dissatisfied	21 (12.7)
Rather dissatisfied	22 (13.3)
Somewhat satisfied, somewhat dissatisfied	50 (30.3)
Rather satisfied	25 (15.2)
Very satisfied	47 (28.5)

* Data based on available medical records. ** Percentages for combined treatment modalities do not sum to 100% as some patients received trimodal therapy.

**Table 2:** Descriptive statistics of well-being and depressive symptoms (*N* = 165).

Scale/Subscale	Min–Max	Mean	SD	Mdn	Mo
KIDSCREEN-10 (Overall, T-score) *	22–49	35.93	5.17	37.00	38 and 39
Satisfaction with Life Domains (SATISF)	14–72	34.61	15.13	30.00	14
CDI-2™ (Total score—T-score) *	18–60	56.06	4.20	56.00	56
Subscale A: Negative mood	17–22	19.93	1.55	20.00	21
Subscale B: Low Self-Esteem/Anhedonia	11–16	13.26	1.27	13.00	14
Subscale C: Ineffectiveness	13–17	15.45	1.25	16.00	16
Subscale D: Interpersonal Problems	5–12	8.02	2.50	7.00	5

M—Mean; SD—Standard Deviation; Mdn—Median; Min–Max—Minimum and Maximum; Mo—Mode. * CDI-2™ T-score interpretation: <60 (Normal/Average), 60–64 (High Average/Subclinical), 65–69 (Slightly Elevated), ≥70 (Very Elevated). KIDSCREEN-10 T-score: 50 ± 10 (Normative Mean); <30 (Clinically Low/Significant Impairment).

**Table 3:** Cluster Centers (standardized values) *.

Variable	Cluster 1 (*N* = 41)	Cluster 2 (*N* = 34)	Cluster 3 (*N* = 45)	Cluster 4 (*N* = 41)	F	*p*	Post Hoc Comparison
Satisfaction with Life Domains (SATISF)	−0.87	−1.10	1.69	1.48	322.84	<0.001	2 < 1 < 3 1 < 4 < 3
CDI-2™ A: Negative mood (Sadness, worry)	0.86	−1.43	−0.61	0.17	72.48	<0.001	2 < 3 < 4 < 1
CDI-2™ B: Low self-esteem (Distorted self-image, anhedonia)	0.41	−0.71	−0.22	0.90	35.46	<0.001	2 < 4 3 < 1 < 4
CDI-2™ C: Ineffectiveness (Feeling of incompetence)	−0.35	−1.65	0.32	0.75	32.96	<0.001	2 < 1 < 4 2 < 1 < 3
CDI-2™ D: Interpersonal problems (Social relationships)	−0.96	1.06	0.31	−0.52	289.74	<0.001	1 < 4 < 3 < 2

* Cluster labels (1–4) reflect predominant functional characteristics observed in the psychometric data; they are intended for descriptive purposes to highlight the heterogeneity of psychological adaptation. A value of 0 represents the mean for the entire study population. Positive values (>0) indicate above-average intensity of a given trait, while negative values (<0) indicate below-average intensity.

**Table 4:** Multinomial logistic regression model parameters for individual clusters (Reference Category—Cluster 4) *.

Independent Variable	Comparative Category	B (SE)	Wald χ^2^	*p*	OR [Exp(B)]	95% CI for OR
Complexity of treatment	Cluster 1 vs. 4	2.19 (0.51)	18.25	<0.001	8.91	3.27–24.31
	Cluster 2 vs. 4	0.53 (0.63)	0.72	0.393	1.70	0.50–5.79
	Cluster 3 vs. 4	−0.11 (0.63)	0.03	0.862	0.90	0.26–3.06
Type of cancer	Cluster 1 vs. 4	−0.88 (0.24)	13.74	<0.001	0.42	0.26–0.66
	Cluster 2 vs. 4	−0.40 (0.21)	3.72	0.054	0.67	0.45–1.01
	Cluster 3 vs. 4	−0.44 (0.24)	3.33	0.068	0.65	0.41–1.03
Quality of life (HRQoL)	Cluster 1 vs. 4	−0.56 (0.11)	27.26	<0.001	0.57	0.46–0.71
	Cluster 2 vs. 4	0.60 (0.16)	14.35	<0.001	1.81	1.33–2.47
	Cluster 3 vs. 4	−0.06 (0.07)	0.91	0.938	0.94	0.82–1.07
Biological Sex	Cluster 1 vs. 4	−0.24 (0.45)	0.28	0.595	0.79	0.33–1.90
	Cluster 2 vs. 4	0.12 (0.47)	0.06	0.807	1.12	0.45–2.82
	Cluster 3 vs. 4	−0.30 (0.56)	0.28	0.599	0.75	0.25–2.23
Chronological Age	Cluster 1 vs. 4	−0.410 (0.12)	11.31	<0.001	0.66	0.52–0.84
	Cluster 2 vs. 4	−0.88 (0.15)	34.53	<0.001	0.41	0.31–0.56
	Cluster 3 vs. 4	−0.35 (0.14)	6.32	0.024	0.70	0.53–0.93

B—Unstandardized regression coefficient; SE—Standard error; Wald χ^2^—Wald chi-square statistic; *p*—*p*-value; OR—Odds ratio [Exp(B)]; 95% CI—95% Confidence interval for OR. * The wide confidence intervals for ‘Complexity of treatment’ reflect the sample size constraints within stratified cluster comparisons; however, the lower bounds remain consistently above 1.0, indicating a stable risk direction.

**Table 5:** Exploratory trend analysis: Parameters of the multinomial logistic regression models for systemic and sociodemographic factors (Reference Category—Cluster 4).

Independent Variable	Comparative Category	B (SE)	Wald χ^2^	*p*	OR [Exp(B)]	95% CI for OR
Age at diagnosis	Cluster 1 vs. 4	−0.48 (0.18)	7.35	0.007	0.62	0.44–0.88
	Cluster 2 vs. 4	0.31 (0.27)	1.31	0.253	1.36	0.80–2.32
	Cluster 3 vs. 4	−0.38 (0.17)	4.87	0.027	0.69	0.49–0.96
Time since treatment completion	Cluster 1 vs. 4	−0.06 (0.17)	0.11	0.745	0.95	0.68–1.33
	Cluster 2 vs. 4	0.03 (0.26)	0.01	0.906	1.03	0.63–1.70
	Cluster 3 vs. 4	0.13 (0.17)	0.60	0.599	1.14	0.82–1.58
Father’s education level	Cluster 1 vs. 4	0.79 (0.39)	4.16	0.042	2.21	1.03–4.73
	Cluster 2 vs. 4	0.18 (0.44)	0.16	0.688	1.19	0.51–2.81
	Cluster 3 vs. 4	−0.31 (0.36)	0.78	0.378	0.73	0.36–1.47
Mother’s education level	Cluster 1 vs. 4	0.09 (0.44)	0.05	0.832	1.10	0.47–2.58
	Cluster 2 vs. 4	1.36 (0.59)	5.27	0.022	3.89	1.22–12.41
	Cluster 3 vs. 4	−0.45 (0.40)	1.30	0.254	0.64	0.29–1.39
Peer relationships (satisfaction)	Cluster 1 vs. 4	−5.90 (1.17)	25.39	<0.001	0.003	<0.01–0.03
	Cluster 2 vs. 4	−0.46 (0.63)	0.53	0.466	0.63	0.18–2.17
	Cluster 3 vs. 4	−4.60 (1.13)	16.57	<0.001	0.01	<0.01–0.09

B—Unstandardized regression coefficient; SE—Standard error; Wald χ^2^—Wald chi-square statistic; *p*—*p*-value; OR—Odds ratio [Exp(B)]; 95% CI—95% Confidence interval for OR.

## Data Availability

The original contributions presented in this study are included in the article. However, due to the sensitive nature of the collected data, the involvement of a vulnerable study population (minors), and restrictions concerning identifiable medical information and patient confidentiality, the raw datasets cannot be made publicly available. Further inquiries and reasonable requests for data access should be directed to the corresponding author.

## Abbreviations

The following abbreviations are used in this manuscript:

ANOVA	Analysis of Variance
CDI-2™	Children’s Depression Inventory–2
CI	Confidence Interval
DFA	Discriminant Function Analysis
GCP	Good Clinical Practice
HRQOL	Health-Related Quality of Life
HSD	Honestly Significant Difference
M	Mean
Mdn	Median
Mo	Mode
OR	Odds Ratio
*p*	*p*-value
SD	Standard Deviation
